# Genomic regions, cellular components and gene regulatory basis underlying pod length variations in cowpea (*V. unguiculata* L. Walp)

**DOI:** 10.1111/pbi.12639

**Published:** 2016-10-17

**Authors:** Pei Xu, Xinyi Wu, María Muñoz‐Amatriaín, Baogen Wang, Xiaohua Wu, Yaowen Hu, Bao‐Lam Huynh, Timothy J. Close, Philip A. Roberts, Wen Zhou, Zhongfu Lu, Guojing Li

**Affiliations:** ^1^ Institute of Vegetables Zhejiang Academy of Agricultural Sciences Hangzhou China; ^2^ State Key Lab Breeding Base for Sustainable Control of Plant Pest and Disease Zhejiang Academy of Agricultural Sciences Hangzhou China; ^3^ Department of Botany and Plant Sciences University of California‐Riverside Riverside CA USA; ^4^ Department of Nematology University of California‐Riverside Riverside CA USA

**Keywords:** Cowpea, Domestication, GWAS, Pod length, Selection, Transcriptome

## Abstract

Cowpea (*V. unguiculata* L. Walp) is a climate resilient legume crop important for food security. Cultivated cowpea (*V. unguiculata* L) generally comprises the bushy, short‐podded grain cowpea dominant in Africa and the climbing, long‐podded vegetable cowpea popular in Asia. How selection has contributed to the diversification of the two types of cowpea remains largely unknown. In the current study, a novel genotyping assay for over 50 000 SNPs was employed to delineate genomic regions governing pod length. Major, minor and epistatic QTLs were identified through QTL mapping. Seventy‐two SNPs associated with pod length were detected by genome‐wide association studies (GWAS). Population stratification analysis revealed subdivision among a cowpea germplasm collection consisting of 299 accessions, which is consistent with pod length groups. Genomic scan for selective signals suggested that domestication of vegetable cowpea was accompanied by selection of multiple traits including pod length, while the further improvement process was featured by selection of pod length primarily. Pod growth kinetics assay demonstrated that more durable cell proliferation rather than cell elongation or enlargement was the main reason for longer pods. Transcriptomic analysis suggested the involvement of sugar, gibberellin and nutritional signalling in regulation of pod length. This study establishes the basis for map‐based cloning of pod length genes in cowpea and for marker‐assisted selection of this trait in breeding programmes.

## Introduction

Cowpea (*V. unguiculata* L. Walp., 2n = 2x = 22), native to Africa, is a worldwide important legume used as a grain, fodder or vegetable crop. Sub‐Saharan Africa and Brazil are the major producers of cowpea grain, while East/South‐East Asia is the main vegetable‐type cowpea producer (Rachie, 1985; Timko *et al*., 2007). Grain‐type cowpea, commonly known as African cowpea or common cowpea (*V. unguiculata* L. Walp. ssp. *unguiculata*), provides a major source of dietary protein for millions of people in developing countries (Singh, 2002). The vegetable cowpea, also known as asparagus bean or ‘yardlong’ bean (*V. unguiculata* L. Walp. ssp. *sesquipedalis*), is characterized by its long tender pods that are harvested when immature, narrow kidney‐shaped seeds, and strong trailing and climbing growth habit that is rare in ssp. *unguiculata* germplasm. The asparagus bean pods can either be snapped and cooked in stew or stir‐fried, or preserved with salt and chile. They provide a good source of proteins, vitamins and minerals (Timko *et al*., 2007). Besides the nutritional value, asparagus bean is among the top ten Asian cultivated vegetables due to its high tolerance to heat and drought (National Research Council, 2006).

Although vegetable cowpea is predominant in East/Southeast Asia, cowpea for grain use is also grown in some parts of these regions. Many of the latter are not typical ssp. *unguiculata* varieties but are landraces of ssp. *sesquipedalis* that can be classified into the ‘nonstandard’ vegetable type based on genetic compositions (Xu *et al*., 2012). In some regions of Africa, Europe and America, vegetable cowpea is also cultivated, although these forms usually exhibit a ‘bush’ phenotype and develop relatively shorter pods than asparagus beans (National Research Council, 2006). To date, it is still unknown how the vegetable and grain cowpea types have diverged. It has been postulated that ssp. *sesquipedialis* was derived from domesticated ssp. *unguiculata* after it was brought to parts of Asia through intense selection for pod characteristics favourable for vegetable use (Fang *et al*., 1996; Kongjaimun *et al*., 2012a,b). In this regard, asparagus bean would have gone through double domestication bottleneck in that only a small portion of the genetic variation present in the progenitor African cowpea germplasm would have constituted the foundation germplasm of ssp. *sesquipedialis* (Fang *et al*., 1996; Timko *et al*., 2007). This theory has been partially validated by the lower genetic diversity in Chinese asparagus bean germplasm compared to African common cowpea through SSR and SNP marker analyses (Xu *et al*., 2010, 2012). However, evidence that selection for pod characteristics, particularly pod length, has contributed essentially to the formation of present‐day asparagus bean is lacking. Also, the relationships between pod length QTLs and other domestication genes/QTLs are not well understood. To our knowledge, the only QTL analysis of pod length in asparagus bean was reported by Kongjaimun *et al*. (2013a) using SSR markers.

The recently developed Cowpea iSelect Consortium Array (Illumina, Inc.) provides an opportunity to further dissect pod length and other domestication traits. This array contains 51 128 SNPs that derive from WGS sequencing of 37 diverse cowpea accessions (33 grain cowpeas and four asparagus bean) (Muñoz‐Amatriaín *et al*., 2016). Using this assay, a cowpea consensus genetic map was constructed with SNP data from five mapping populations (Muñoz‐Amatriaín *et al*., 2016). In the current study, we describe the application of this assay to dissect pod length QTLs in cowpea. In addition, histological and gene expression analyses were performed to uncover the cellular components and gene regulatory basis underlying pod length variations.

## Results

### SNP genotyping and data curation

**Figure 1:**
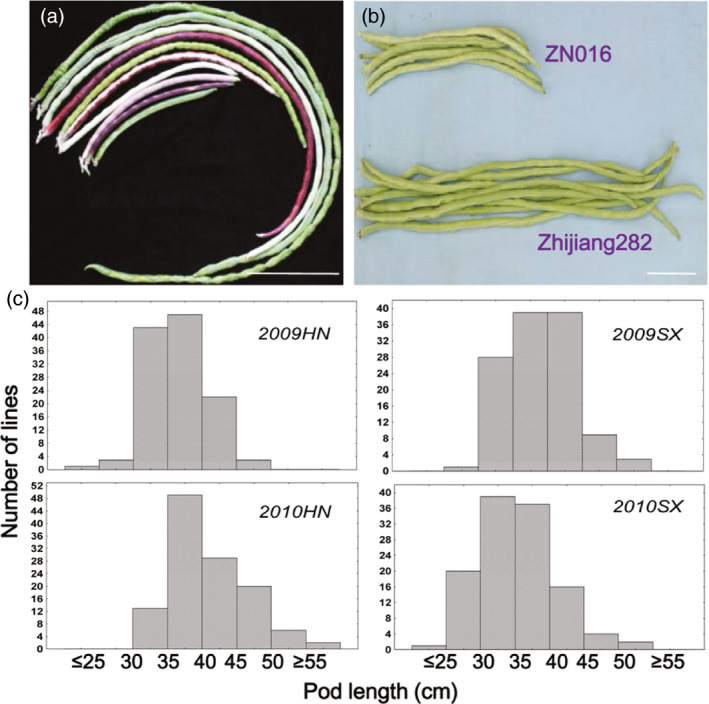
Pod length phenotypes. (a) Variation of pod length among selected germplasm lines. (b) Pod morphology of ZN016 and Zhijiang282, the parents of the RIL population. (c) Distribution of pod length in the ZN016× Zhijiang282 RIL population. Number of RILs = 119. Scale bar = 10 cm.

We SNP genotyped 432 DNA samples including 132 recombinant inbred lines (RILs) and a diversity panel of 299 landraces, cultivars and breeding lines (Figure 1a, b; Table S1). A total of 49 194 SNPs produced successful calls. After the removal of samples showing high heterozygosity, missing data or nonparental alleles (for the RIL population), 119 RILs and all germplasm accessions were used for further analysis. After eliminating monomorphic SNPs and those with excessive number of missing and/or heterozygous calls and very low minor allele frequencies, 7988 high‐quality SNPs were retained for the RIL population and 30 211 for the diversity panel.

### Phenotypic analysis of pod length

Zhijiang282, the male parent of the RIL population, exhibited longer pods (mean = 47.0 cm) than the female parent ZN016 (mean = 31.9 cm) in all experiments. Among the RILs, pod length displayed a continuous distribution with the population means falling between the parental values (Figure 1c). Transgressive segregation was observed as some RILs exhibited a pod length outside the parental value range, suggesting the existence of intragenic (e.g. incomplete dominance or co‐dominance) or intergenic interactions. High correlation (0.7–0.82, *P *<* *0.001) was observed for pod length between different trials. The estimated broad‐sense heritability was 70.9% over the 2 years. A wider range of variation (12.3–74.5 cm) and a continuous distribution based on multiyear pod length data were observed for the diversity panel. Correlation coefficients between experiments ranged from 0.87 to 0.93 (*P *<* *0.001) for this population.

### iSelect genetic map construction and QTL mapping

At a LOD score of 10 for marker grouping, 7964 of the 7988 SNPs (99.7%) were mapped to 697 bins in 11 linkage groups (LGs) (Figure S1). This map, hereafter referred to as ‘ZZ map v.2’ in consistency with its earlier version (Xu *et al*., 2011a), covered 803.4 cM with an average distance between marker bins of 1.15 cM and an average density of 11.4 SNPs per bin. The length of individual LGs varied from 45.1 cM to 124 cM, and the average marker distance per LG ranged from 0.05 cM to 0.16 cM (Table S2). The map contained two gaps of >10 cM, located on LG1 and LG5, respectively.

**Figure 2:**
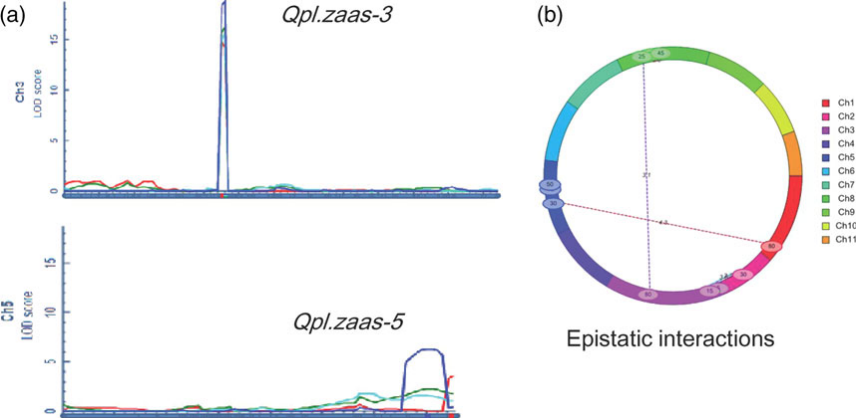
QTLs detected under the additive (a) and epistatic interaction (b) models implemented in QTL iCIMapping software. In (a), different colours represent results obtained from different trials. Dark blue: 2009HN; Red: 2010HN; Green: 2010SX; Light blue: 2009SX. Number of RILs for mapping = 119.

Inclusive composite interval mapping identified two major QTLs for pod length under the additive model (ICIM‐ADD) (Figure 2a, Table 1). The larger effect QTL, designated as *Qpl.zaas‐3*, was mapped within a 1.3‐cM interval on LG3 between the markers *2_04960* and *2_02274* in all four experiments. The LOD scores in different experiments varied from 14.7 to 18.7, and the average phenotypic variance explained was 44%. A significant interaction of *Qpl.zaas‐3* with environment was also detected (Table 1), but its effect on phenotypic variance was small (8%). Another QTL spanning a 9.5‐cM interval on LG5 (*Qpl.zaas‐5* hereafter) was detected at a LOD score above 3 in the 2010HN trial, and above LOD = 6 in the 2009HN trial. It explained 11% of the phenotypic variance on average. Genome‐wide scan of digenic interactions identified four pairs of epistatic interactions, three interchromosomal and one intrachromosomal (Figure 2b). The phenotypic variance explained by the individual pairs of epistatic interactions ranged from 6.4% to 18.2%. No common epistatic interactions were detected across all four environments, and none of the loci involved in those epistatic interactions coincided with *Qpl.zaas‐3* or *Qpl.zaas‐5*.

**Table 1:** QTLs detected with a LOD score ≥3 under the ICIM‐ADD model

QTL	LG	Position (cM)	Marker interval	Single environment				LOD.^c^A×E
				Environment	LOD	aPVE%	bAdditive	
*Qpl.zaas‐3*	3	44.8‐46.1	*2_04960‐2_02274*	2009HN	18.7	45.7	−3.8547	4.4
				2009SX	15.4	45	−3.398	
				2010HN	14.7	39.1	−2.6831	
				2010SX	16.1	46	−3.6866	
*Qpl.zaas‐5*	5	66‐75.5	*2_12600‐1_1130*	2009HN	6.3	13.7	−2.053	
				2010HN	3.6	7.8	−1.1896	

a PVE, phenotypic variation explained. Average proportion of variation explained.

b The negative additive effects indicated that ZN016 contributed the allele to a decrease in pod length.

c A by *E* effect, the additive and dominance × environment effect.

### Genetic diversity and population structure of the germplasm panel

**Figure 3:**
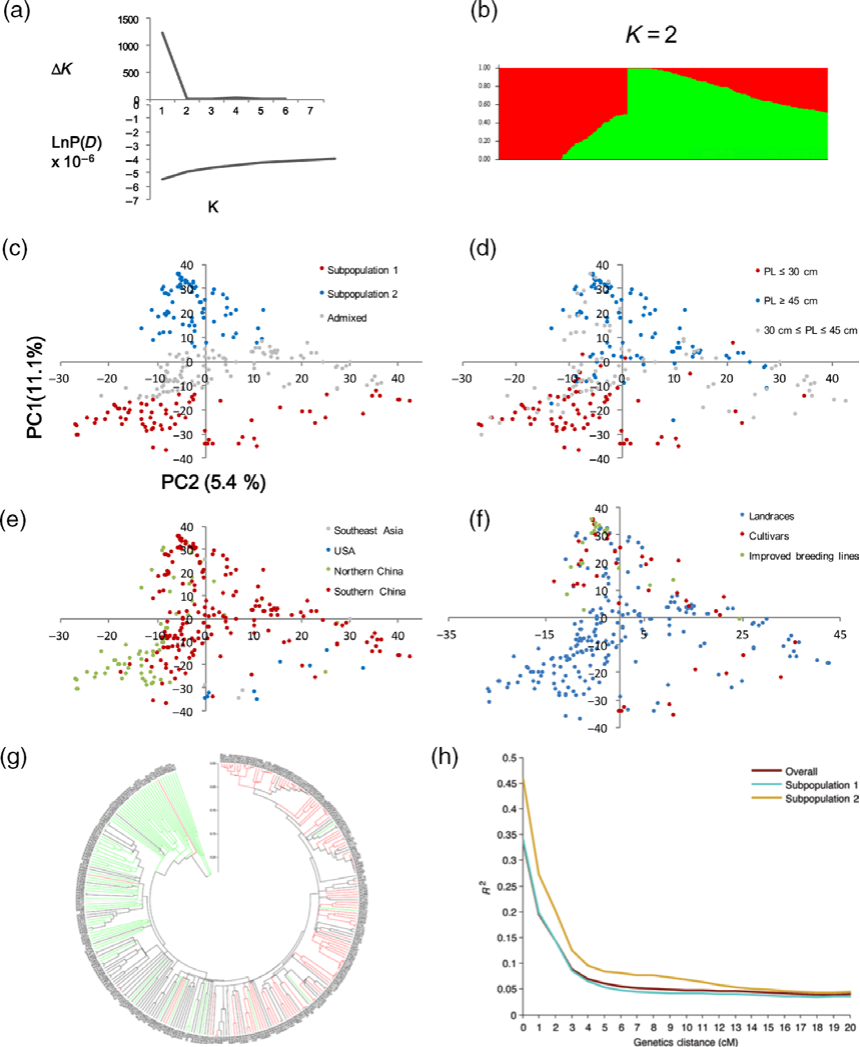
Subpopulation inference, principal component analysis (PCA) and dendrogram of the germplasm collection (299 accessions). (a) A plot of LnP(D) and delta K against K. Note that the scales for *Y*‐axis are not proportional above and under zero; (b) estimated population structure of the germplasm collection inferred at *K* = 2; c–f, display of PCA results with the accessions coloured by population subgrouping (c), pod length group (d), geographic origin (e) or breeding status (f); (g) an unrooted phylogenetic tree showing the dendrogram of all samples. Accessions with pod length shorter than 30 cm are marked in red, longer than 45 cm in green and between 30 and 45 cm in black; h, decay of linkage disequilibrium (LD) in all samples, subpopulation 1 and subpopulation 2. The strength of LD was measured by *r* ^2^.

The overall heterozygosity rate of the germplasm panel estimated with 30 211 high‐quality SNPs was 2.23%, fitting the expectation for inbred plant materials. Pairwise genetic distances between the 299 accessions ranged from 0.0003 to 0.5556, with a mean of 0.253. A model‐based clustering method identified two major subpopulations in the germplasm panel (Figure 3a, b, Table S1). Subpopulation 1 consisted of 79 accessions, including all nine American accessions, three of the four Filipino accessions and 67 Chinese accessions. The mean and median pod length in this subpopulation were 26.1 cm and 25.6 cm, respectively. All members of this subgroup except for seven Chinese accessions and two American accessions are grain types, and all the Chinese accessions are landraces. Subpopulation 2, consisting of 99 accessions with 98 from China and one from Thailand, had a mean and median pod length of 53.1 cm and 53.4 cm, respectively. All accessions from this subpopulation were typical vegetable cowpea accessions. The rest of the accessions with the probability of belonging to a subpopulation lower than 0.7 were grouped as ‘admixed’. Principal component analysis (PCA) supported the subgroup assignment of model‐based method in that the accessions assigned to subpopulations 1, 2 and the admixed group, in particular subpopulation 1, were well distinguishable along the first PC (*Y*‐axis, Figure 3c). The PCA result also revealed that the two subpopulations were mainly differentiated by pod length rather than by geographic origin or improvement status (Figure 3d, e, f), reinforcing the critical role of pod length in the diversification of cowpea. An unrooted neighbour‐joining tree constructed for subpopulation 1 and subpopulation 2 accessions showed a clear (albeit not perfect) branching between the short‐podded and long‐podded types (Figure 3g, Figure S2).

### Genome‐wide association studies (GWAS) for pod length

**Figure 4:**
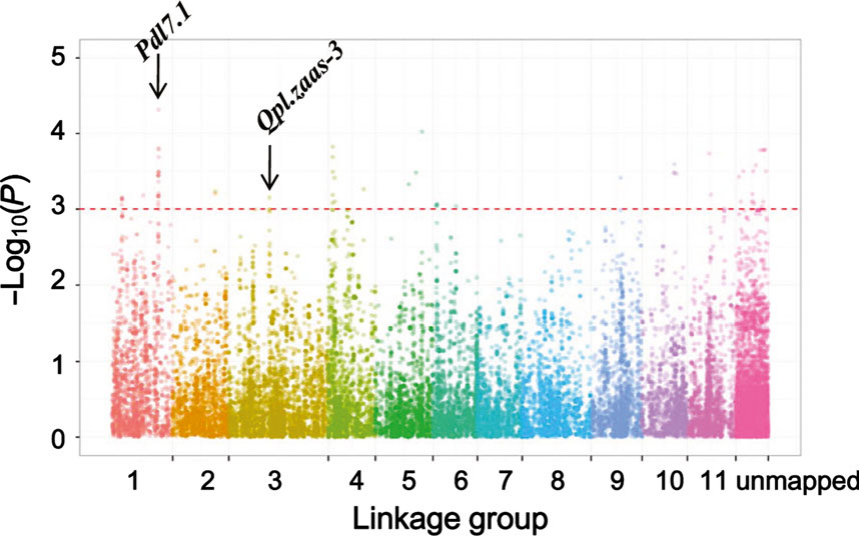
Manhattan plot displaying significant associations between SNP markers and pod length. The position of the major QTL *Qpl.zaas‐3* detected in single‐family QTL analysis in this study and the position of the major QTL *Pdl7.1* reported in Kongjaimun et al. (2012a) are indicated with black arrows. The horizontal axes indicate the consensus map position of each SNP while the vertical axes indicate the ‐log10 of the *P*‐values. The dash line indicates the threshold of 3. The false discovery rate (FDR) for significant SNPs is listed in Table S3.

Prior to GWAS, the extent of LD was analysed for the whole set of accessions and for each of the subpopulations. The LD decay (*r* ^2^ = 0.2) with genetic distance occurred at ~2 cM across the whole panel. LD decayed (cM values) faster in subpopulation 1 than in subpopulation 2 where more breeding lines were included (Figure 3h). GWAS was performed for pod length using a mixed‐linear model (MLM) method correcting for population structure and kinship. We identified 72 SNP loci associated with pod length, of which 55 had known map positions (Figure 4, Table S3). The 55 SNPs were distributed among nine of the 11 LGs, representing 16 SNP clusters. Observed phenotypic variation explained by a single SNP varied from 4.6% to 7.1%. The two significant SNP makers on LG3 coincided with *Qpl.zaas‐3*, which spans the region 51.87–54.99 cM on the cowpea consensus map (Muñoz‐Amatriaín *et al*., 2016).

### Population genome‐wide scan for selection signals

**Figure 5:**
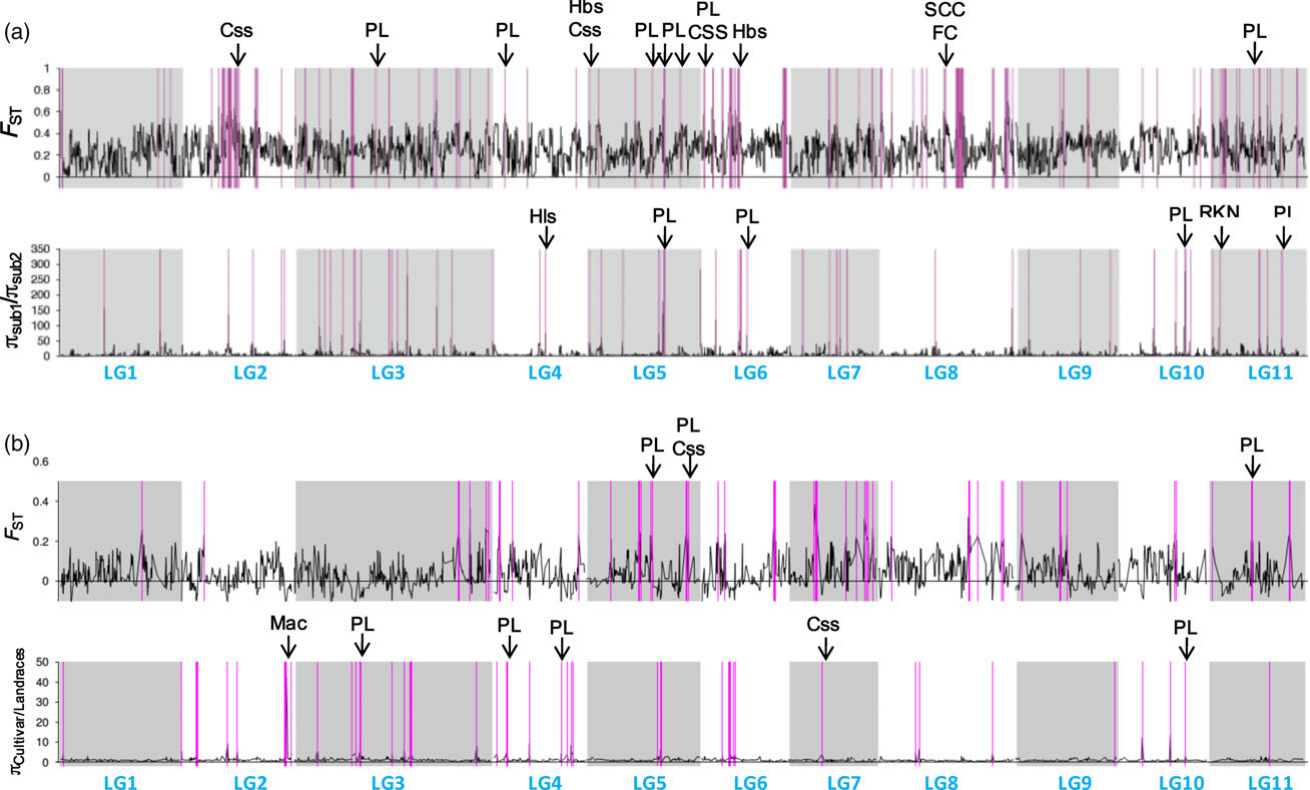
Genome scan for *F*_ST_ and π ratio. For (a), the parameters were calculated between the two subgene pools and for (b) between the cultivars/breeding lines and landraces of subpopulation 2. Both parameters were first calculated at each SNP site and then were averaged and plotted via a kernel‐smoothing moving method that took 0.15 cM sliding windows with 0.03 cM steps to generate genome‐wide distributions. A bootstrap resampling technique was applied for assigning significance threshold values. One million replicates were run for each statistic. Putative selective signal regions (outliers of *F*_ST_ and π ratio, *P *≤* *0.05) are highlighted in purple. Qualitative genes/QTLs overlapping with the selective signals are marked with black arrows. PL: pod length; Hbs: heat‐induced brown discoloration of seed coats; RKN: resistance to root‐knot nematodes; SSC: seed coat colour; FC: flower colour; Css: seed size; Hls: hastate leaf shape; Mac: resistance to *Macrophomina phaseolina*.

A genome‐wide scan of population differentiation index (*F*_ST_) and nucleotide diversity (π) was carried out first between the two subgene pools and then between the landraces and cultivars/breeding lines from subpopulation 2. Overall *F*_ST_ between subpopulation 1 and subpopulation 2 was 0.262 (Table S4), indicating a moderate population divergence. The mean π value in subpopulation 1 was 0.314, more than twice that of in subpopulation 2 (0.13, Table S4), suggesting a severe loss of genetic diversity in the vegetable cowpea subgene pool. A fine‐scale inspection of π and *F*_ST_ in a window sizing 0.15 cM (roughly 100 Kb of physical distance) with a step size of 0.03 cM across the genome revealed variations among and fluctuations across LGs, as expected. A total of 40.5 cM of differentiated genomic regions as indicated by *F*_ST_ outliers were determined between the two subgroups (*P *≤* *0.05, Figure 5a, Table S5), accounting for 4.6% of the total length of the genetic map. Selective sweeps as suggested by nucleotide diversity ratio (π_subpopulation1/subpopulation2_) outliers were detected in genomic regions totalling 15.33 cM in length, representing ~1.8% of the genome (*P *≤* *0.05, Figure 5a, Table S5). When comparing the locations of these regions with 42 reported SNP‐tagged qualitative genes/QTLs governing domestication/resistance traits (Table S6) and 55 pod length‐associated SNPs identified in this study, many of them were found to be overlapping (Figure 5a). Given the very small portion of the genome tagged as diverged and selected regions, the coincidences are unlikely to occur by chance alone. Among these overlapping regions, ten were related to pod length. The remaining seven regions harbour genes/QTLs governing many other traits such as seed size, seed coat colour, flower colour, leaf shape and root‐knot nematode resistance. As the two subgene pools under investigation are predominantly comprised of grain and vegetable cowpeas, respectively, our results may suggest that domestication of the vegetable cowpea was accompanied by selection for both pod length and other domestication/resistance traits.

*F*_ST_ and π ratio outliers between landraces and cultivars are considered to be indicative of selective signals during crop improvement after domestication (Shi and Lai, 2015). Our *F*_ST_ outliers analysis between landraces and cultivars/breeding lines in subpopulation 2 revealed a total of 16.32 cM of differentiated genome regions (*P *≤* *0.05, Figure 5b). Selective sweeps (π_cultivars/_π_landraces_ outliers) were detected in genome regions totalling 13.74 cM in length. Seven of these regions were found to be coincident with pod length QTLs, and only three with QTLs controlling other traits. These results suggest that pod length appears to be the primary selection target during improvement of vegetable cowpea after its domestication. Among the seven regions overlapping with pod length QTLs, two were specific to the improvement process.

### Growth kinetics and histological analysis of the long and short pods

**Figure 6:**
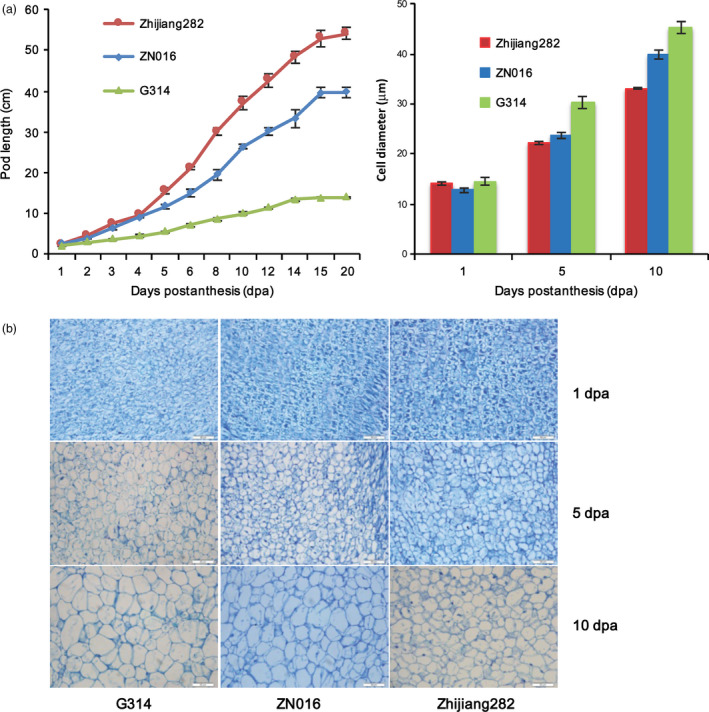
Growth kinetics and cellular morphology of sectioned pods in three genotypes. (a) kinetics of pod elongation after anthesis (left panel) and cell diameters measured at different dpa, *n* = 20 (right panel); (b) longitudinally sectioned pod cells at different dpa. Error bars indicate SE. Scale bar = 50 μm.

The genotypic differences of pod length might result either from different cell size or from cell number. To elucidate this, cell diameters were measured for sectioned pods among three genotypes representing the long‐ (Zhijiang282), medium‐ (ZN016) and short‐podded (G314) genotypes, respectively. Their growth kinetics after anthesis are shown in Figure 6a. We found that Zhijiang282 reached a stable pod length much later than G314, with ZN016 being intermediate. This indicates more durable pod growth activity in Zhijiang282. At the early stage of postanthesis (1 dpa) during pod elongation, the cell diameters were similar among genotypes (Figure 6b, c). At the later stages (5 dpa and 10 dpa), Zhijiang282 did not show more elongated or enlarged cells, but rather had a similar or even smaller cell sizes than the shorter‐pod lines. This phenomenon was explained by the slower growth rate of pods in Zhijiang282 and suggested that the eventually long‐pod phenotype in Zhijiang282 was due primarily to more durable cell proliferation rather than to cell elongation/enlargement.

### Comparative transcriptomic analysis between the long‐ and short‐pod pools

Custom microarray hybridizations of a long‐pod and a short‐pod pool were performed to reveal the gene regulatory basis underlying pod length differences. Three technical replicates were included and showed a high consistency between each other (pairwise correlation coefficients ranged from 0.931 to 0.997, Figure S3). A total of 873 and 1228 genes were found to be more abundantly expressed in long‐pod and short‐pod pools, respectively (fold change ≥2, FDR ≤ 0.05, Table S7). *SWEETIE*, encoding a glycosyl transferase known to involve in the regulation of sugar flux (Veyres *et al*., 2008), was found among the top differentially expressed genes (DEGs). It suggests that sugar signalling is involved in pod length regulation. Gene ontology (GO) enrichment analysis of the DEGs revealed that GO terms including ‘mitotic cell cycle process’, ‘cytokinesis by cell plate formation’ and ‘cyclin‐dependent protein serine/threonine kinase regulator activity’ were significantly enriched (Table S8). This conformed to the results of histological analysis and suggests a role of cell division in pod length regulation. Additionally, the enrichment of ‘gibberellin mediated signalling pathway’, ‘nutrient reservoir activity’ and ‘response to carbon dioxide’ indicates that hormonal and nutritional signalling are also important mechanisms related to pod length control.

## Discussion

The past decade has witnessed significant advances in genome research for cowpea including asparagus bean (e.g. Kongjaimun *et al*., 2012a,b; Muchero *et al*., 2009; Xu *et al*., 2012). Here, we used the Cowpea iSelect Consortium Array to genotype a RIL population and a large association panel for asparagus bean to map QTLs controlling pod length. Our results, in line with previous studies (Kongjaimun *et al*., 2012a; Vidya *et al*., 2002), show a high heritability of pod length in asparagus bean, suggesting that the use of marker‐assisted selection (MAS) in the improvement of this trait in early generations is feasible. Consistent also with previous studies (Hazra *et al*., 2007; Rashwan, 2010), we found that additive effects serve as the major genetic basis for pod length. In particular, the ‘one major QTL + minor QTLs’ mode of pod length determination as discovered in our intervarietal population is similar to the case in an asparagus bean × grain cowpea population (Kongjaimun *et al*., 2012a). It is interesting to note that in some other related *Vigna* species such as azuki bean (Isemura *et al*., 2007), rice bean (Isemura *et al*., 2010) and mung bean, a similar mode of pod length inheritance was reported. Nevertheless, the extremely broad variability of pod length has been only observed in cowpea. This implies the occurrence of species‐specific mutations in pod length genes in *V. unguiculata*. By incorporating a broader range of genetic variations available in the germplasm collection, the GWAS analysis uncovered additional QTLs associated with pod length. *Qpl.zaas‐3*, the major QTL detected in the biparental RIL mapping study, was also detected by GWAS. Due to a shared SSR marker (cp07863) between the earlier version of the ZZ map (Xu *et al*., 2011b) and the Kongjaimun *et al*. (2012a) genetic map, we found that the major QTL *Pdl7.1* reported in Kongjaimun *et al*. (2012a) was coincident with a cluster of significant SNPs on LG1 in our study (Figure 4). Some other highly significant SNP clusters (e.g. on LG4, 5, 10 and 11) may represent novel pod length QTLs. Besides additive gene effects, epistasis as part of the genetic components affecting pod length was also disclosed, suggesting the feasibility of utilizing heterosis in breeding programmes targeting pod length.

It is a common phenomenon that QTLs governing different domestication traits are linked or are involved in pleiotropic effects. In cowpea, tightly linked or pleiotropic QTLs were reported for pod length, plant height, seed size, flowering time, seed coat colour, flower colour and other traits (Peksen, 2004; Ubi *et al*., 2000; Xu *et al*., 2011b, 2013). Among the QTLs detected in our population, *Qpl.zaas‐3* overlaps a previously mapped major QTL for pod number per plant (*Qpn.zaas‐3*, Xu *et al*., 2013), which could explain the long‐standing findings of strong correlation between the two traits (Aggarwal *et al*., 1982; Bapna *et al*., 1972). Recently, extensive co‐localizations of QTLs controlling pod tenderness and pod length were also reported (Kongjaimun *et al*., 2013), pointing to the co‐domestication of agricultural traits in vegetable cowpea.

**Figure 7:**
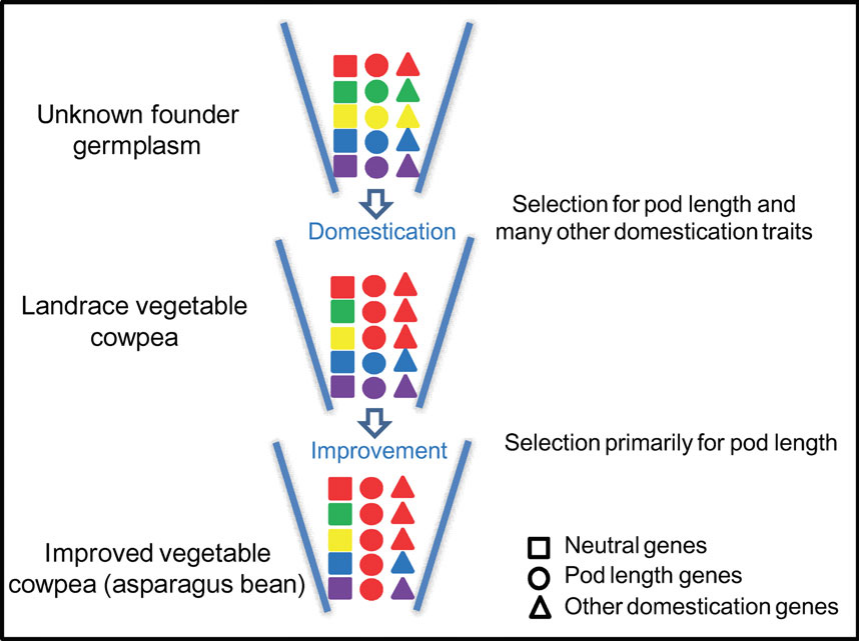
A graphical model depicting the signatures of natural or artificial selection driving the diversification of grain and vegetable cowpea. Different colours represent genetic diversity. We assume that the founder germplasm of vegetable cowpea was naturally or artificially selected for many domestication and resistance traits including pod length; the domesticated vegetable cowpea may have then undergone further selections towards pod length primarily, leading to the present‐day asparagus bean.

Population structure analyses suggested a general division of the grain cowpea and vegetable cowpea subgene pools among the investigated diversity panel. Based on comparative analysis of known QTLs and putative selective signals during vegetable cowpea domestication and improvement, we were able to tentatively propose a model for the diversification of grain and vegetable cowpea (Figure 7). We assume that founder germplasm of vegetable cowpea, after being introduced into the place of their domestication in Asia, was naturally or artificially selected for both pod length and many other domestication/resistance traits important to its adaptation to the local target agro‐ecosystem. The long‐lasting wet season in Asia must have favoured vegetative growth, and thus late maturing and pest/disease‐resistant cowpea became more adapted. The domesticated landrace vegetable cowpea may have then undergone further intensive selections towards increased pod length, leading to the present‐day asparagus bean. This may partly be owing to the fact that cowpea varieties with longer soft pods are more attractive to local consumers and growers due to cooking convenience and higher yield contribution (Umaharan *et al*., 1997). During this process, severe loss of genetic diversity would have occurred, resulting in the low genetic diversity in asparagus bean. Hence, restoring specific beneficial or favourable alleles in the current asparagus bean varieties and breeding lines may require introgression from landrace asparagus bean lines or grain‐type cowpeas.

Our study provides novel insights into the diversification of cowpea selected for vegetable and grain uses. Nevertheless, several caveats should be mentioned regarding the interpretation of our results due to technical limitations. First, we used a fixed SNP chip (albeit high genome coverage and density) for genotyping which may suffer from ascertainment bias (Albrechtsen *et al*., 2010). However, asparagus beans were included in the SNP discovery panel (Muñoz‐Amatriaín *et al*., 2016). Also, the impact of a possible ascertainment biases on our results may be low because one of the key statistics, *F*_ST_, used in this study was reported to be ‘tolerant’ to ascertainment bias (Albrechtsen *et al*., 2010). Second, the uneven distribution of the markers on the genetic map and co‐segregation of physically distant loci may confound the results. Therefore, drawing a more conclusive picture of the domestication history of vegetable cowpea will benefit from whole genome (re‐)sequencing studies. In the near future, a complete cowpea reference genome is expected to enable identification of causal genes from the QTL regions. The analysis of pod development and its transcriptional regulations conducted in this study will be of particular use for such task as it provides biological information complementary to genetic analyses. Another interesting future work is to measure the dynamic behaviour of pod length for all individuals from the mapping population. These data, when integrated into a functional mapping framework (Wu and Lin, 2006), will provide new insights into the developmental genetic mechanisms of pod length.

## Experimental procedures

### Plant materials and growth conditions

The population used in genetic mapping included 132 recombinant inbred lines (F_6:8_, ‘ZZ’ population) developed by single‐seed descent from the cross ‘ZN016’ × ‘Zhijiang282’. ‘ZN016’ is a landrace vegetable cowpea accession originating from southern China with medium‐long pods (~38 cm) while ‘Zhijiang282’ is a typical asparagus bean cultivar with long pods (~55 cm, Figure 1a). Field experiments were carried out in two consecutive years (2009 and 2010), each in two locations, that is Haining County (HN, 30°32′N, 120°41′E) and Shaoxing County (SX, 29°43′N, 120°14′E). Each experiment had two replicates, except for SX2009 with one replicate. Eight to ten seeds per line were sown every 28 cm in 25‐m‐long plots on rows 75 cm apart, but only four uniform seedlings were retained per line after seedling emergence due to the size and trailing habit of the adult plants. The plots were spaced by 50 cm to avoid border effect. A set of 299 cowpea accessions, either grain or vegetable type, was used as the diversity panel (Table S1, Figure 1b). The accession ‘G93’ was sampled twice in order to provide an internal control of data quality, leading to a total sample number of 300. The plants were grown in HN in 2014 and 2015 for pod length measurement, with two replicates in each experiment. Growth conditions and field management were similar to those for the RIL population.

### DNA extraction, SNP genotyping and raw data processing

Genomic DNA was extracted from leaves of field‐grown plants 1 month after sowing using DNeasy Plant DNA miniprep kits (Qiagen, Hilden, Germany). Standardized DNA for the samples, the majority at a concentration of 50 ng/μL and a small fraction 10–30 ng/μL were hybridized to the Cowpea iSelect Consortium Array according to the standard protocol. Single‐base extension was performed and the chips were scanned using the Illumina iScan. Image files were saved for cluster file analysis. The clustering algorithm of GenomeStudio Genotyping Module (V 1.9.4, Illumina, Inc.) was used for SNP calling. To increase the accuracy of the clustering algorithm, additional samples including synthetic heterozygous were added to the workspace. Polymorphic SNPs were identified based on Illumina GenTrain score (proximity of clusters) and call frequencies across samples. All markers were manually inspected and curated based on manufacturer's best‐practice instructions. All marker sequences can be retrieved from the HarvEST database (http://harvest.ucr.edu) and detailed information from Muñoz‐Amatriaín *et al*. (2016).

### Linkage mapping

Linkage mapping of the SNP markers was performed with MSTmap (Wu *et al*., 2008). Before computing the marker orders and distances, SNPs were filtered using the following criteria: missing call rate ≤20% (equivalent to a minimum effective population size of 106), heterozygous call rate ≤10% and minor allele frequency (MAF) ≥0.25. Critical parameters for mapping were as follows: population type = RIL at generation 6; no. of mapping size threshold = 2; no. of mapping distance threshold = 15 cM; no. of mapping missing threshold = 25%; genetic mapping function = Kosambi; try to detect genotyping errors = no.

### Phenotyping

Pod length was phenotyped by measuring the distance from the peduncle connection point to the apex of the pod. Ten representative pods were measured per line, and the data were averaged.

### QTL mapping

QTL IciMapping V4.0 (Wang *et al*., 2014) was used to perform inclusive composite interval mapping (ICIM‐ADD) for pod length. Critical mapping parameters were as follows: step size = 1 cM, PIN = 0.001. A LOD threshold of three was used to determine major QTLs. The ICIM‐EPI function implemented in the software was used to scan for QTL interactions with a LOD threshold of three. QTLs × environment interactions were detected under the ‘multi‐environmental trials’ mode with a LOD score threshold of three. QTLs detected in different environments within same, adjacent or overlapping marker intervals were designated as the same.

### Diversity, population structure and kinship analyses

Pairwise genetic distances were calculated using TASSEL 5.1 (Bradbury *et al*., 2007) based on 30 211 SNPs, which was a subset of the 49 194 technically successful SNPs after filtration with the following criteria: missing call rate ≤20%, heterozygous call rate ≤20% and MAF ≥ 0.01. Population structure was inferred using a two‐step procedure. Firstly, we ran the software STRUCTURE 2.3.4 (Pritchard *et al*., 2000) under the ‘admixture model’ with a burn‐in period of 100 000 followed by 100 000 replications of Markov chain Monte Carlo with 2320 SNPs each with a discrete map position on the latest cowpea consensus map (Muñoz‐Amatriaín *et al*., 2016). Five independent runs each were performed with the number of clusters (K) varying from 1 to 8. The Evanno method (Evanno *et al*., 2005) was used to determine the optimal K for subgrouping. Next, the software FastStructure (Raj *et al*., 2014) that enables time‐efficient handling of a large number of markers was used to run all the 30 211 SNPs at the optimal K to give the whole genome ancestry estimates (Q‐matrix). Lines with a probability of membership ≥70% were assigned to a subgroup (otherwise, ‘admixed’). Relative kinship matrix was generated using the same set of 30 211 SNP markers with TASSEL 5.1. The neighbour‐joining tree was generated by MEGA5 based on genetic distance data (Tamura *et al*., 2011). LD was measured by calculating the square value of correlation coefficient (*r* ^2^) between each SNP pair.

### Genome‐wide trait–marker association study (GWAS)

A trait–marker association analysis was performed using Tassel 5.1 under a mixed‐linear model (MLM) that corrects for both population structure (Q) and relative kinship (K). The phenotypic data used were a combined set of field data from multiple experiments. Significant SNPs were defined if showing a minus log10‐transformed *P *≥* *3. The FDR multiple test correction was performed using the software QVALUE (Storey, 2002). SNPs with a genetic distance less than 2 cM were considered to be in a LD extension block and belong to the same SNP cluster.

### Population genomic parameters calculation and scanning across the genome

Nucleotide diversity (π) was calculated using the formula $\pi =1-\sum_{i}\left(\begin{array}{c} n_{i}\\ 2 \end{array}\right)/\left(\begin{array}{c} n\\ 2 \end{array}\right)$, where *n*_*i*_ denotes the count of allele *i* and *n *= Σ *n*_*i*_. Population differentiation index (*F*_ST_) was estimated adapting the formula $F_{\mathrm{ST}}=1-\frac{\sum_{j}\left(\begin{array}{c} n_{j}\\ 2 \end{array}\right)\pi_{j}}{\pi \cdot \sum_{j}\left(\begin{array}{c} n_{j}\\ 2 \end{array}\right)}$ that accounts for unbalanced population sizes according to Nielsen *et al*. (2009). These parameters were first calculated at each of the 25 873 SNP site mapped onto the cowpea consensus map and then were averaged and plotted via a kernel‐smoothing moving method that took 0.15 cM sliding windows with 0.03 cM steps to generate genome‐wide distributions. With the estimated cowpea genome size of 630 Mb and the consensus genetic map length of 837.11 cM, the window and the sliding step size roughly equalled 100 Kb and 20 Kb of physical distances, respectively. Wherever the π value as denominator was zero, the corresponding *F*_ST_ value was replaced with the genomic mean. The method for assigning significance threshold values was based on a bootstrap resampling technique as described in Hohenlohe *et al*. (2010). One million replicates were run for each statistic.

### Comparative analysis of known genes/QTLs and putative selective signals

Map position information of known qualitative genes or QTLs governing domestication and resistance traits in cowpea was assembled from a literature search. Only genes/QTLs tagged by SNP markers that are comparable to our marker system were used in the analysis. SNP markers reported to be tightly linked to qualitative genes or located around the peak regions of QTLs were extracted. A coincidence between a known gene/QTL and a selective signal was declared if the SNP markers fell into the outlier window regions of *F*_ST_ or π ratio. If the known QTLs (or single associated SNPs) were detected by GWAS, their coincidence with a selective signal was determined by the overlap of the LD block where the SNP resides (2 cM downstream and upstream of the significant SNP) and the window region of the selective signal.

### Pod growth kinetics assay, paraffin sectioning and histological analysis

Pod samples collected at 1, 5 and 10 dpa were fixed in FAA (5 mL 38% formalin: 5 mL glacialacetic acid: 90 mL 70% alcohol) for 24 h and then dehydrated in a graded ethanol series, substituted with 1‐butanol and embedded in Paraplast Plus. The samples were sectioned longitudinally at 4 μm thick using a rotary microtome (RM2135,LEICA). The sections were stained with methylene blue and observed under a light microscope (BH2, OLYMPUS). For cell diameter comparison, ten cells from each sample were measured and the data averaged.

### Custom cDNA microarray construction, hybridization and data processing

A custom Agilent microarray targeting 29 471 cowpea unigenes was transferred from a previous Roche NimbleGen cowpea microarray (Xu *et al*., 2015). Hybridizations were performed for long‐pod pools and short‐pod pools, each consisting of RNA from seven independent genotypes, in three replicates. Details of the 14 genotypes used for pooling can be found in Table S1. Methods for RNA extraction, purification and quantification were as described in Xu *et al*. (2015). All cDNA were labelled with the fluorescent dye Cy5‐dCTP using cRNA amplification and labelling kit (CapitalBio, Beijing, China). Array hybridization, washing and image scanning were conducted according to the manufacturer's instructions. The raw data were summarized and normalized using the GeneSpring software V12.0 (Agilent). Differentially expressed genes (DEGs) were defined by an expression fold change ≥2 between the two pools and a corrected *P*‐value (Benjamini‐Hochberg FDR) ≤0.05. Hierarchical clustering was performed with Cluster 3.0 using the average linkage method, and the results were visualized using TreeView (Eisen *et al*., 1998).

### Gene ontology (GO) enrichment analyses

Gene ontology (GO) enrichment analyses were performed using GOrilla (Eden *et al*., 2009) under a *P*‐value threshold of 10E^−3^ for statistical significance. The GObase used was the version updated on 5 December 2015. Prior to running GOrilla, the cowpea unigenes were BLASTx searched against the Arabidopsis protein sequences database under a *P*‐value cut‐off of 10E^−5^ to generate legible sequence IDs for recognition in the program. The entire list of unigenes on the chip was inputted as the background for calculation.

## Data availability

All the raw data used in this study are publicly available. The Cowpea iSelect Consortium Array is available from Illumina (Illumina Inc., San Diego, CA; http://www.illumina.com/areas-of-interest/agrigenomics/consortia.html). Source data for microarray experiment were deposited in the NCBI Gene Expression Omnibus under the GEO series accession number GSE80552.

## Supporting information


**Figure S1** Graphic representation of the “ZZ” genetic map v.2. Each horizontal line represents a bin.

**Figure S2** An unrooted Neighbor‐Joining phylogenetic tree of all samples. Accessions with pod length shorter than 30 cm are denoted as S, longer than 45 cm as L, and between 30 and 45 cm as M following the accessions IDs.

**Figure S3** Microarray expression profiles of cowpea genes in the long‐pod and short‐pod pools. The log‐transformed values of relative expression levels were used for cluster analysis.

**Table S1** Germplasm lines used in the current study. Pod length data are the mean of three trials from 2014 to 2015 in Haining, China.

**Table S2** Marker positions on the ZZ genetic map v.2.

**Table S3** SNP markers associated with pod length as detected by GWAS.

**Table S4** Nucleotide diversity and population differentiation index values at each mapped SNP.

**Table S5** *F*_ST_ and π ratio outliers across the genome (*P *≤* *0.05)

**Table S6** Qualitative genes/QTLs or trait‐associated SNPs used for comparison with diverged regions and putative selection signals across the genome.

**Table S7** Genes that were differentially expressed between the long and short‐podded pools. In red are genes more abundantly expressed in long pods and in green are genes more abundantly expressed in short pods. FC=fold change.

**Table S8** List of the GO terms overrepresented between the two RNA pools.
