# Supplementary material for: Genomic regions, cellular components and gene regulatory basis underlying pod length variations in cowpea (V. unguiculata L. Walp)
Source: Plant Biotechnol J. 2016 Oct 17;15(5):547–57. doi: 10.1111/pbi.12639 (PMC5399003; doi:10.1111/pbi.12639)
Supplement: Supplementary file 1 — Figure S1 Graphic representation of the “ZZ” genetic map v.2. Each horizontal line represents a bin. [file PBI-15-547-s005.docx]

**Fig S1. Graphic representation of the “ZZ” genetic map V2.** Each horizontal line represents a bin.

LG
