# Supplementary figures and images for: Genomic regions, cellular components and gene regulatory basis underlying pod length variations in cowpea (V. unguiculata L. Walp)
Source: Plant Biotechnol J. 2016 Oct 17;15(5):547–57. doi: 10.1111/pbi.12639 (PMC5399003; doi:10.1111/pbi.12639)

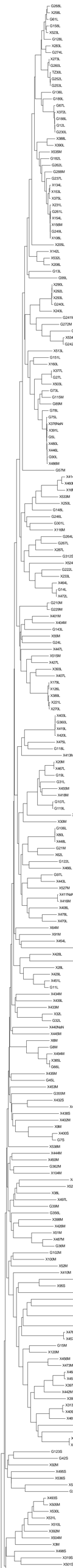

Supplement: Supplementary file 2 — Figure S2 An unrooted Neighbor‐Joining phylogenetic tree of all samples. Accessions with pod length shorter than 30 cm are denoted as S, longer than 45 cm as L, and between 30 and 45 cm as M following the accessions IDs. [file PBI-15-547-s009.pdf]
