# Supplementary material for: Genomic regions, cellular components and gene regulatory basis underlying pod length variations in cowpea (V. unguiculata L. Walp)
Source: Plant Biotechnol J. 2016 Oct 17;15(5):547–57. doi: 10.1111/pbi.12639 (PMC5399003; doi:10.1111/pbi.12639)
Supplement: Supplementary file 3 — Figure S3 Microarray expression profiles of cowpea genes in the long‐pod and short‐pod pools. The log‐transformed values of relative expression levels were used for cluster analysis. [file PBI-15-547-s008.docx]

**Fig. S3. Microarray expression profiles of cowpea genes in the long-pod and short-pod pools.** The log-transformed values of relative expression levels were used for cluster analysis.
